# A Method for Growing Bio-memristors from Slime Mold

**DOI:** 10.3791/56076

**Published:** 2017-11-02

**Authors:** Eduardo Reck Miranda, Edward Braund

**Affiliations:** ^1^Interdisciplinary Centre for Computer Music Research, Plymouth University

**Keywords:** Bioengineering, Issue 129, Memristor, *Physarum polycephalum*, 3D printing, bioware, wetware, biological computing, biocomputer music

## Abstract

Our research is aimed at gaining a better understanding of the electronic properties of organisms in order to engineer novel bioelectronic systems and computing architectures based on biology. This specific paper focuses on harnessing the unicellular slime mold *Physarum polycephalum* to develop bio-memristors (or biological memristors) and bio-computing devices. The memristor is a resistor that possesses memory. It is the 4th fundamental passive circuit element (the other three are the resistor, the capacitor, and the inductor), which is paving the way for the design of new kinds of computing systems; *e.g.*, computers that might relinquish the distinction between storage and a central processing unit. When applied with an AC voltage, the current vs. voltage characteristic of a memristor is a pinched hysteresis loop. It has been shown that *P. polycephalum* produces pinched hysteresis loops under AC voltages and displays adaptive behavior that is comparable with the functioning of a memristor. This paper presents the method that we developed for implementing bio-memristors with *P. polycephalum* and introduces the development of a receptacle to culture the organism, which facilitates its deployment as an electronic circuit component. Our method has proven to decrease growth time, increase component lifespan, and standardize electrical observations.

**Figure:**
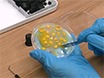


## Introduction

Today’s computers are built using the three two-terminal fundamental passive circuit elements: the capacitor, the resistor, and the inductor. Passive elements are only capable of dissipating or storing energy, not generating it. These elements were established in the 18^th^ and 19^th^ century and are linked through Maxwell’s equations. We define each of these three circuit components in terms of their relationship between two of the four circuit variables namely, current (I), voltage (V), charge (Q), and flux-linkage (φ). The charge is the time integral of the current and Faraday’s law defines the voltage as the time integral of the flux. Thus, a capacitor is defined by a relationship between voltage and charge, a resistor is defined by a relationship between voltage and current, and the inductor is defined by a relationship between flux and current. For well over a century, these elements were a cornerstone of electronics. However, they only represent three of the possible four relationship pairs between the circuit variables, leaving flux-linkage and charge unlinked. In 1971, Leon Chua published a paper^1^ where he postulated that there was a missing fourth element that linked the remaining two variables, which he called the memristor. The memristor can be described as a resistor that remembers its history, hence the contraction 'memory resistor.' This element functions by altering its resistance according to the magnitude of the previously applied voltage and its duration. Moreover, the memristor retains its last resistance state once the voltage is no longer applied. Unlike the capacitor, resistor, and inductor, the memristor's behaviour is nonlinear, which is evident in its I-V profile where a pinched hysteresis loop is formed under an AC voltage. This loop takes the form of a Lissajous figure containing two perpendicular oscillations of high and low resistant states. Before Chua’s formalized memristance theory, other researchers had reported on memory resistance effects at certain frequencies when experimenting with materials such as polymers and metal oxides, along with developing electrical devices at the micrometer scale^2^. However, in many cases, these effects were considered undesirable. It took almost forty years for Chua’s formalization to be connected to a physical device and for researchers to begin developing methods of exploiting memristive effects. A team at the HP Laboratories succeeded in fabricating a memristive device in 2008^3^ that ignited huge interest in the element. 

Computer scientists have a keen interest in the memristor due to it being credited as the first element to combine processing and memory abilities in a single unit. It also displays behaviors that are analogous to certain neurological processes such as Spike-Timing-Dependent Plasticity (STDP)^4^, to name but one. Such behaviors are giving rise to perspectives of building brain-like computing technologies that relinquish the distinction between memory and central processing unit (or CPU)^5^. In contrast to the popular approaches to developing memristors (using TiO_2_, for example), our ambition is to develop an organic bio-memristor. Furthermore, we are interested in how this component may provide means of exploring paradigms beyond conventional approaches to engineering computing devices; *e.g.*, creative applications in the field of Computer Music^6^.

Memristance is an effect that researchers have recently found throughout a range of biological systems. For example, memristive properties have been observed in aloe vera plants^7^ and human skin^8^, to cite but two. These discoveries indicate that it may be possible to implement processing and memory devices on biological substrates. Harnessing organic systems within technology may allow us to explore exciting concepts such as self-assembly, self-repair, low environmental impact, and self-powering. Before we can investigate these opportunities however, several challenges need to be addressed. Many of the biological systems that have memristive properties have significant constraints that limit their viability as an actual electronic component. For example, an aloe vera leaf^7^ needs light, has a limited lifespan, and would be difficult to integrate into a circuit. Furthermore, several other *in vivo* memristive phenomena, such as human sweat ducts^8^, are not currently feasible options for developing systems for use outside of the laboratory and in everyday electronic systems. However, of all the memristive phenomena, there is one potential candidate: *P. polycephalum*.

The plasmodium of *P. polycephalum* is an amorphous unicellular system that has been discovered to act as a memristive component^9^,^10^. This organism is an ideal candidate for research in hybrid hardware-wetware electronics for a number of reasons. Firstly, the organism is non-pathogenic, macroscopic, and requires no specialist equipment use, which renders the plasmodium accessible to engineers and non-biologists. Secondly, the cell is amorphous, forms networks of wire-like veins, and will grow on most substrates (**Figure 1**). These properties allow the cell's morphology to be easily delineated to conform to a conventional electrical scheme. There is also research demonstrating that the plasmodium can live for over four years^11^, and that its veins can act as self-repairing conductive pathways^12^. Several laboratory studies have confirmed the organism's memristive abilities^9^,^10^,^13^ and now the time is ripe to explore its potential.

The idea of using *P*. *polycephalum* memristors is relatively new. As a result, there are no established standards for measuring and observing its electrical properties. Such a lack of uniformity in experimental procedures within the same research group and between groups may be the reason there are inconsistencies amongst published results^9^,^10^. It is likely that such variation is most prominent in sample growth conditions and handling. Thus, we need to establish methods for producing and testing *P. polycephalum* memristors where factors that might cause errors are better controlled and monitored.  Furthermore, we need to create methods of implementing *P. polycephalum* memristors that allow for stable and easy integration into electrical systems.

The method presented in this paper provides a platform for exploration of practical applications of *P. polycephalum *memristors by providing means of incorporating the organism as a component into an electrical schematic. It is likely that these techniques will appeal to engineers looking to explore real-world uses of hybrid hardware-wetware systems. Furthermore, it is accessible to non-experts (*e.g.*, open-source electronic prototyping enthusiasts) who may be interested in experimenting with aspects of unconventional computing but have found it difficult to find prototypes to adapt to their needs. Some potential applications may include implementing probabilistic models harnessing the memristors spiking behavior, developing approaches to performing stateful logic operations, and modelling neurological processes for information storage and processing.

## Protocol

### 1. Fabrication of a 3D Printed Receptacle

1. **Chambers, lids, and base**
  1. Load a 3D printer with High-Impact Polystyrene (HIPS) by using the printer interface to set the print bed temperature to 85 °C and the extruder to 230 °C. When the temperatures are reached, loosen the idler arm, insert the filament, and push down until it starts to extrude out of the hot end. Then, retighten the filament idler arm and remove the extruded material.
  2. Import the 3D receptacle STL model file into a 3D printing slicing software, which can normally be achieved by navigating to the file tab and selecting the import/open options (Figure 2).
  3. If slicing software offers high and low quality print settings, select high quality while also ensuring that the correct material profile is selected. NOTE: If printing several receptacles in one run, make sure that the software is set to print each object one at a time. If this step is skipped, the print quality may be reduced, which will likely cause tolerance issues when fitting the parts together.
  4. Once the printing is complete, wait until the print bed temperature is below 50 °C to remove the parts.
  5. Using a thin wire brush, gently clear the electrode socket of any imperfections that may cause obstructions when fitting the chamber with an electrode.
2. **Electrodes**
  1. Replace the HIPS filament for a cleaning filament and run plenty of the material through the print head.
  2. Load the printer with an electrically conductive polylactic acid (PLA) filament that has a volume resistivity of 0.75 Ω-cm or less.
  3. Set the print bed temperature to 60 °C and the extruder to 230 °C (see step 1.1.1 for guidance).
  4. When the temperatures are reached, extrude several centimeters of the filament through the print head. This process will help ensure that all particulates from previous sessions are removed.
  5. Using a 3D printing slicing software, load the electrode STL file (Figure 3).
  6. In the print settings, specify the following: Layer height = 0.16 mm, Shell thickness = 1.7 mm, Bottom/Top thickness = 0.74 mm, Fill density = 100% (Figure 4).
  7. If printing several electrodes in one run, set the printer to print one at a time.
  8. Once printed, leave the electrodes on the print bed until they have cooled to room temperature. This ensures the part does not become warped and misshaped.
3. **Receptacle assembly**
  1. Slot an electrode into each of the two chambers. If step 1.1.5 has been completed correctly, the electrodes should go into the chambers without much force.
  2. Using a sharp scalpel, cut a 10 mm piece of Polyvinyl chloride (PVC) tubing (4 mm inner diameter and 6 mm outer diameter) taking care to ensure that each end is cut straight and cleanly.
  3. Gently ease each end of the 10 mm PVC tubing over the rim of the two electrodes.
  4. Once connected, the two chambers clip into the base.

### 2. Receptacle Preparation and *P. polycephalum* Inoculation

1. **2% agar medium preparation**
  1. Put 2 g of non-nutrient microbiological agar powder into a 250 mL glass bottle.
  2. Add 100 mL of deionized water and mix well.
  3. Autoclave the bottle for 12 - 15 min at 121 °C or place in a boiling water bath for 15 - 20 min.
2. **Setting the agar substrate into the receptacle's chambers**
  1. Melt the agar using a water bath or microwave.
  2. Fill a 2 mL pipette with molten agar.
  3. Fill each of the receptacle's chambers by hovering the nib of the pipette approximately 5 mm above the internal base and slowly filling the wells up to the bottom of the connecting tube hole.
  4. Immediately after filling the wells, place a lid on each of the chambers and set the receptacle aside until the agar has set and reached room temperature.
3. ***P. polycephalum* inoculation**
  1. Place an oat flake in each of the two chambers.
  2. Remove one 2 mL blob of pseudopods from a starved (approximately 12 h) culture of plasmodium and place it in one of the two chambers. To promote speedy growth, try to take the protoplasm from the most active anterior of the organism.

## Representative Results

To produce representative results, we set up 5 samples using the exact method described above. For a control, 5 samples were also arranged using the method described in the early *P. polycephalum* memristor investigations^9^,^10^. Here, we positioned two electrodes spaced at a distance of ~10 mm within 60 mm Petri dishes. Each electrode consisted of a circle (~20 mm in diameter) of tinned copper wire (16 stands at 0.2 mm) filled with a 2% non-nutrient deionized agar (~2 mL). All samples were monitored via time-lapse imagery to review growth time. Here, the 5 receptacle samples connected the two electrodes within 10 h of inoculation. The fastest of these grew in under 2 h, and the longest was 10 h, with the mean average growth time across all 5 samples of 7 h 24 min. Four of the control samples produced a linking protoplasmic tube and one propagated off the inoculation electrode but dried out before it made the required connection. The quickest of the control samples made its connection within 19 h while the slowest took 36 h, with an average growth time across control samples of 26 h 15 min. These data show a significant decrease in growth time for memristors grown using the presented method.

The I-V profile of a memristor is its most defining feature. As such, we performed I-V measurements on the samples to produce representative results for this paper. Here, instantaneous current measurements were made at each point of a 160-step voltage sine wave. Each voltage step had a static dwell time of 2 s. Electrical measurements were made using a 230 Programmable Voltage Source and a 617 Programmable Electrometer. These devices were selected as they are capable of sourcing voltage and taking measurements at high resolutions. Experiments were conducted at room temperature in an unlit room.

Figure 6 shows typical I-V curves produced from tests on *P. polycephalum* memristors. Figure 6c and **6d** show plots with the representative measurements from components implemented in the Petri dishes. Results using this method show that, although curves measured on the same sample are morphologically similar, hysteresis varies heavily from sample-to-sample. Such variation includes the location of pinch points, the magnitude of both positive and negative lobes, and the symmetry between measurements in the negative and positive voltage domains. Thus, I-V curves measured on memristors using the Petri dish method are not the footprint of an ‘ideal’ memristor because pinch points are not at zero voltage and current. Figure 6a and **6b** show graphs with representative measurements from memristors grown in the receptacles. The pinch point locations and lobe sizes of these hysteresis loops are relatively consistent both in discrete sample curves tested under different voltage ranges and time-steps, and sample-to-sample curves. Therefore, receptacle I-V curves were more reminiscent of an ‘ideal’ memristor's footprint, where pinch points were always singular and almost consistently at zero voltage and current. However, although hysteresis morphologies were similar sample-to-sample, there was variation in overall resistance between samples. .

After the initial I-V measurements were completed, tests were made on each sample once a day until they presented no memristive curves. Of the 4 control samples, 2 dried up within 2 days of initial testing, while the remaining 2 continued to record pinched curves for a further 2 days. The receptacle samples maintained their memristance for at least 7 days, with 3 samples exceeding that. Over time, each of the receptacle sample's protoplasmic tubes became thicker, and there was a decrease in overall resistance, with some samples measuring in the A x 10^-04^ range for 10 V runs against A x 10^-05^ in their earlier tests.

The reader is referred to the article by Braund^14^ for results on the extensive testing of the presented receptacle.

**Figure:**
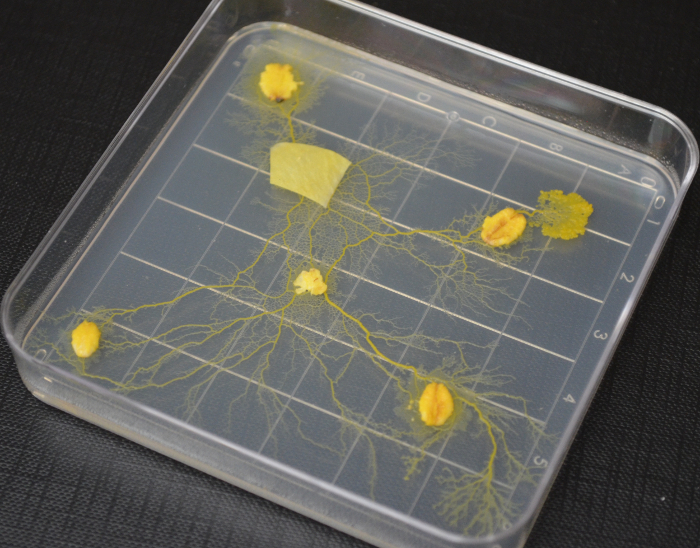

**Figure 1:** **A photograph of a 2 day-old culture of plasmodium of *P. polycephalum*.** Please click here to view a larger version of this figure.

**Figure:**
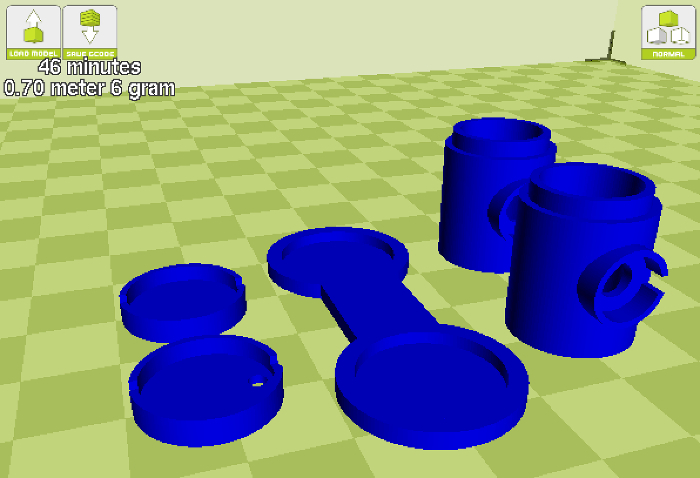

**Figure 2:** **A screenshot of the receptacle STL file after it is loaded into the slicing software.** Please click here to view a larger version of this figure.

**Figure:**
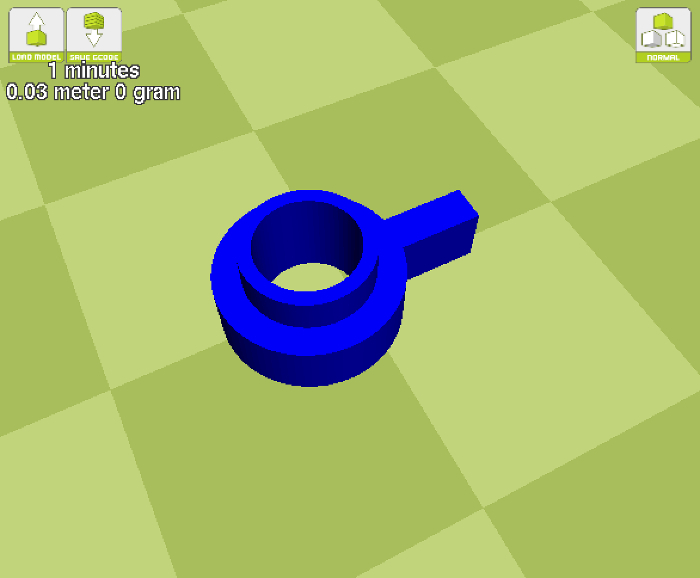

**Figure 3: A screenshot of the electrode STL file after it is loaded into the slicing software.** Please click here to view a larger version of this figure.

**Figure:**
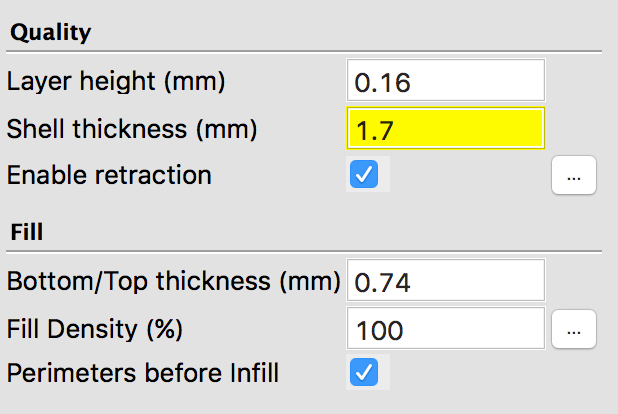

**Figure 4: A screenshot of the settings configuration for printing the electrode STL model.**

**Figure:**
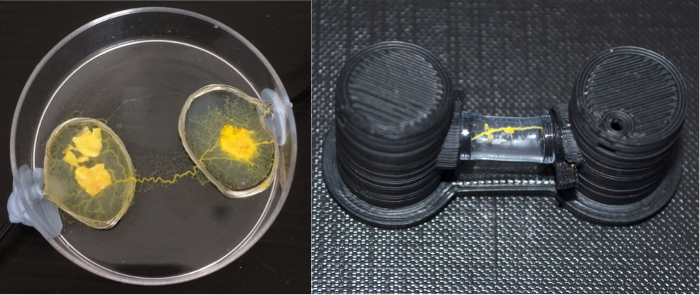

**Figure 5:** **Two photographs depicting *P. polycephalum *memristors implemented in a Petri dish (left) and using the method presented in this paper (right).** Please click here to view a larger version of this figure.

**Figure:**
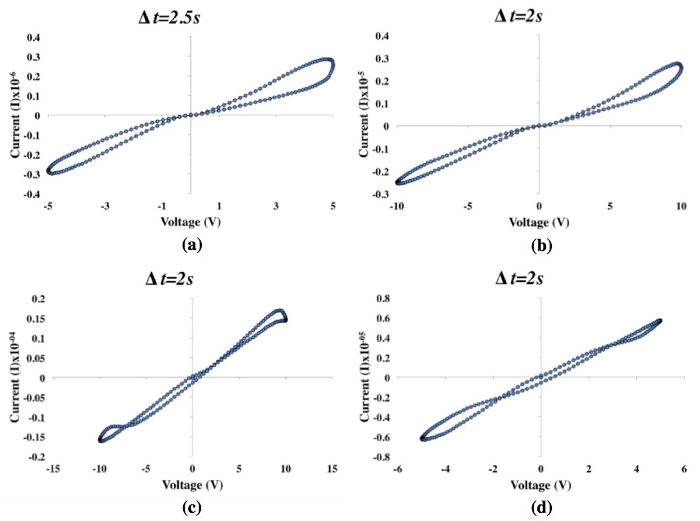

**Figure 6:** Four I-V graphs that were produced from two memristors grown in the receptacles (**a, b**) and two implemented in Petri dishes (**c, d**). Please click here to view a larger version of this figure.

**Figure:**
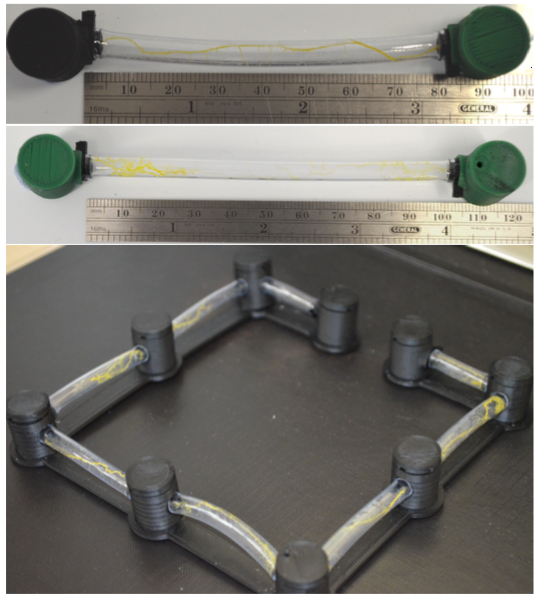

**Figure 7:** **Photographs showing the receptacle being used to grow tubes at various lengths.**

**Figure:**
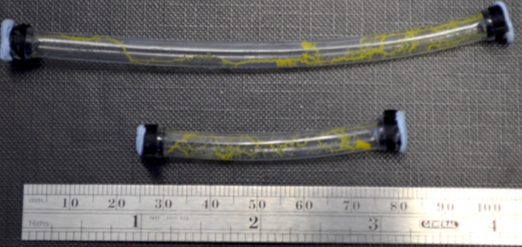

**Figure 8:** **A photograph showing protoplasmic tubes that have been disconnected from the chambers.**

## Discussion

This paper presented a method for growing memristors out of the myxomycete *P. polycephalum*. The organism is grown inside 3D printed receptacles that were designed to overcome some of the constraints that are associated with implementing the bio-memristors. Such limitations include setup time, sample growth time, and lack of standardization for sample-to-sample growth conditions and electrical observations.

Our receptacle was first revealed in 2015 in the printed publicity material for the Peninsula Arts Contemporary Music Festival 2016 (PACMF) and respective website^15^. Here, our technology was used to develop a hybrid hardware-bioware interactive music system that was capable of generating musical accompaniments to a live musician. In reference^14^, we reported on the extensive testing of our receptacle and compared the results against previous approaches^9^,^10^. Following these developments, another group of researchers subsequently explored creating growth environments to study the organism's thermistive properties^16^, but these are not the same as memristive properties. There have, however, only been two other attempts at developing a controlled approach to implementing *P. polycephalum *memristors^13^,^17^. In these experiments, wells were made from a gel-like biocompatible elastomer material called polydimethylsiloxane (PDMS), and electrodes were created using various metals or poly(3,4-ethylenedioxythiophene):poly(styrene sulfonate) (PEDOT:PSS). Although these materials are routinely used in electronics, microfluidics, and bionic engineering, they are expensive and require some expertise to use. For example, PEDOT:PSS needs spin-coating and doping to improve its conductivity. Therefore, the techniques are out of reach for people who do not have access to specialist resources. The receptacles presented in this article use methods and materials that are easily accessed and inexpensive. Furthermore, the design provides a hospitable environment for the plasmodium to habitat, which is in contrast to the other *P. polycephalum *memristor prototypes where no attempt was made to keep the cell alive for any duration of time.

Until now, it has been difficult to obtain consistent I-V measurements using previous methods for culturing the organism on Petri dishes (Figure 5, left). Our methods improved this scenario significantly (Figure 6). The results of our receptacle's testing have demonstrated that the design has decreased growth time, increased lifespan, standardized component responses, and created a protected microenvironment to encapsulate the organism. Furthermore, the device provides feasible means of integrating the organism as a component of an electrical scheme.

The presented method alleviates a number of issues related to harnessing *P. polycephalum *memristors within electrical systems. There are, however, limitations that require further research and development. Firstly, condensation can gather on the connecting tube's inner surface if the receptacles are subjected to a quick change in temperature or if a high voltage is applied for long durations. The latter is due to the organism's high resistance causing electrical energy to be transferred into heat. If significant, the condensation can create a low resistant pathway between the two electrodes at either end of the connecting tube. This limitation can be managed effectively by ensuring that the memristors are not overloaded. Secondly, the overall resistance of memristors produced using the presented method can vary from component-to-component. Such a phenomenon may be a result of the approach not restricting the outer diameter of the protoplasmic tube. Consequently, users may need to incorporate a calibration process into their application of the memristors.

Thanks to this methodology, we can now begin to study the biological processes that are causing memristive observations in *P. polycephalum*. It is likely that such processes have dynamic parameters that we might be able to exploit to augment the element's usage. We have begun running some preliminary experiments where extracellular ion concentrations are altered to review if voltage-gated ion channels play a role in memristance.

The presented receptacles were designed solely for implementing *P. polycephalum* memristors. These devices are likely, however, to have uses beyond that of implementing a single component. For example, in references^12^,^18^, the protoplasmic tube was studied as a self-assembling and self-repairing biological wire. In both these investigations, the researchers expressed that further work was needed to establish methods of growing the protoplasmic tube according to a scheme. The receptacles put forward in this paper provide a method of delineating the production of the tube between two, or potentially more, points. Figure 7 shows two photographs illustrating that the receptacles can be used to grow healthy tubes at lengths more than 100 mm. In reference^18^, the transfer function of the protoplasmic tube was investigated. Results from this investigation indicated that the agar required to grow the tubes may cause an issue if the organism was to be integrated into an electrical system. This is due to the substrate's capacitance. The receptacles presented here still require agar to keep humidity high. However, with small changes to the receptacle's design, it is possible to create a detachable tube. This set up may allow for the tube to be disconnected from the chambers once growth is complete and clipped into an electrical system. Furthermore, once the tube's health begins to deteriorate, it could be reconnected to new chambers for food and respite until it has repaired itself and can be used again. Figure 8 shows a photo of long tubes that have been disconnected from the chambers. Future research is needed to investigate the protoplasmic tube's electrical properties without the agar and when grown at lengths using the presented method.

## Disclosures

The authors have nothing to disclose.
